# Assessment of Odor Removal in Rigid Polypropylene Waste: Comprehensive Characterization and Washing Comparison

**DOI:** 10.1002/gch2.202500567

**Published:** 2026-01-31

**Authors:** Tiago G. A. Belé, Martijn Roosen, Helene M. Loos, Steven De Meester, Andrea Buettner

**Affiliations:** ^1^ Chair of Aroma and Smell Research Department of Chemistry and Pharmacy Friedrich‐Alexander‐Universität Erlangen‐Nürnberg (FAU) Erlangen Germany; ^2^ Laboratory for Circular Process Engineering (LCPE) Department of Green Chemistry and Technology Faculty of Bioscience Engineering Ghent University ‐ Campus Kortrijk Kortrijk Belgium; ^3^ Fraunhofer Institute For Process Engineering and Packaging IVV Freising Germany

**Keywords:** circular economy, deodorization, gas chromatography, plastics recycling, sensory analysis

## Abstract

Control of odor‐active compounds in polymers is fundamental for both recycling and the circular economy. Among deodorization strategies for post‐consumer plastics, washing plays a central role. This study focuses on the odor characterization of rigid polypropylene (PP) waste and the evaluation of different washing procedures for deodorization efficiency. Three washing regimes are tested, varying in temperature (25°C vs. 80°C) and medium (water vs. caustic soda with detergent). The resulting sample sets are classified as unwashed (UW), room temperature washed (RT), hot water washed (HW), and detergent washed (DW). Odor profiles are determined by descriptive sensory analysis, while odor‐active compounds are identified using gas chromatography‐olfactometry (GC‐O). Ranking of odor contributions is performed through odor extract dilution analysis (OEDA). A total of 32 odorants are detected, of which 30 are identified via 2D gas chromatography–mass spectrometry/olfactometry (2D‐GC‐MS/O). Washed material exhibits flowery and soapy impressions, whereas unwashed PP is characterized by moldy notes. Hedonic ratings are lowest for UW, with statistically significant improvement observed in DW. Intensity ratings do not differ significantly across UW, RT, and DW, ranging from 7.5 to 5.5 on a 0–10 scale. These findings link chemo‐sensory methods with odorant removal efficiency, advancing deodorization approaches for plastics.

## Graphical abstract

This study examines how washing modifies odors in post‐consumer polypropylene plastics. Different washing conditions are compared by using sensory panels and advanced chemical analysis. Hot and detergent‐based washing removes key odor‐causing compounds, replacing moldy notes with cleaner, soapy impressions. The results show how targeted washing steps may enhance recycling potential and plastics circularity.

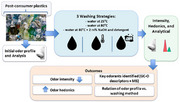

**Abbreviations**
2D‐GC‐MS/O: two‐dimensional gas chromatography‐mass spectrometry/olfactometry
DW: detergent‐washed samples
FID: flame ionization detector
HW: hot water‐washed samples with water at 80°C
GC‐MS: gas chromatography‐mass spectrometry
GC‐O: GC with olfactometry
HDPE: high‐density polyethylene
cOEDA: comparative odor extract dilution analysis;
OD: odor dilution
PE: polyethylene
PP: polypropylene
RT: room‐temperature washed samples with water at 25°C
UW: unwashed samples
VOC: volatile organic compound

## 1 Introduction

Plastics, with their many advantages, are omnipresent in today's society, and plastic production continues to increase further. In 2022, global plastics production reached approximately 390 million metric tons [1, 2]. Projections indicate that, without significant policy interventions, this figure could rise to 736 million metric tons by 2040, reflecting a 70% increase from 2020 levels [2].

In an effort to close the plastic loop, recycling processes are essential to achieve a Circular Economy for plastics [3]. To enable recycled plastics to be fully suitable for packaging, the recycling industry needs to achieve high‐quality final products. One of the main obstacles in recycling is the fact that the final recyclate frequently exhibits, at times, considerable smell nuisances, leaving the recycled polymer no appropriate option in case of demanding applications, such as packaging [4, 5].

To improve odor removal and consequently the smell properties of the final products, several steps along the recycling process, such as washing and degassing, are well established in the recycling industry [3, 6]. For example, in industrial mechanical recycling, washing typically consists of sequential steps involving shredding, friction or flotation washing, hot‐water or alkaline washing, and rinsing [7, 8]. Common operating parameters include water temperatures ranging from 20°C to 90°C, residence times between 5 and 30 min, and agitation via high‐shear friction washers or drum systems [7, 8]. Surfactants or caustic solutions (typically 0.5 wt.%–2 wt.% NaOH) are frequently added to improve the removal of organic residues [7, 8]. One or two rinsing stages are normally performed using ambient water to remove remaining detergents. These conditions are not fully standardized and are often adjusted depending on feedstock composition (e.g., packaging type, expected contamination levels, food vs. cosmetic content) and desired recyclate purity [8]. Accounting for the lack of standardization and to develop and evaluate such processes, it is essential to characterize their efficiency in odor removal both by sensory methods and additionally understand the underlying physical and chemical causative phenomena. In this respect, a number of studies were conducted to elucidate the chemical structures of odor‐active compounds in plastic waste [9, 10, 11] and to establish suitable methods for determining the efficiency of odor removal [12, 13, 14]. However, existing research generally investigates individual washing parameters in isolation, and few studies directly contrast different washing schemes while linking changes in odor profile to specific odorants. This gap motivates a closer examination of washing approaches in post‐consumer polyolefins.

More specifically, with regard to washing processes, researchers evaluated the effect of hot water washing for deodorizing post‐consumer LDPE bags, observing a positive effect toward deodorization [9]. In another study, by analyzing VOC's in general, researchers evaluated the deodorization efficiencies for different post‐consumer plastic packaging waste utilizing different washing processes [15]. In this endeavor, it is, however, essential to prove sensory impact by means of chemo‐sensory analysis, as many VOC compounds are not necessarily odorants. An example of a study included human sensory analysis and focused on washing PP packaging with hot water (80°C) [16]. The researchers demonstrated the deodorization of the polymer material, with odor intensities declining from 7.4 to 4.0 on a scale of 0–10 [16]. The study, however, did not evaluate other washing media.

Thus, a holistic study is needed that analyses odor removal from rigid PP using several different washing media, accompanied by an evaluation by chemo‐sensory analysis. Thus, the present study focuses on:

1. Comprehensive odor profiling of PP waste before and after three different washing conditions: (A) water at room temperature (25°C), (B) hot water (80°C), and (C) a caustic solution with detergent (80°C).
2. Evaluating and identifying the odor‐active compounds in unwashed and washed plastics using comparative odor extract dilution analysis (cOEDA), and (2D) gas chromatography‐mass spectrometry/olfactometry (2D‐GC‐MS/O).
3. Evaluating the washing efficiency regarding odor profile, overall intensity, and hedonic ratings from sensory analyses executed by a trained panel.

## 2 Materials and Methods

### 2.1 Sample Description

An amount of 10 kg of PP waste, mostly composed of bottles (PP copolymers), was sampled at a Belgian waste management company. Plastic packaging products were sampled from the conveyor belt after sorting in an MRF (materials recovery facility). Afterward, plastics were shredded in the lab using a Piovan‐type RSP15/30 shredder with a sieve diameter of 8.0 mm to obtain more homogeneous samples and stored following the protocol described by Roosen and colleagues [15] for two days.

### 2.2 Washing Procedures

Batch‐washing experiments were performed on unwashed (UW) PP waste. The plastic material was washed with different washing media in a 2 L round‐bottom flask. The tested washing media were water at 25°C (room temperature—in the following: RT), water at 80°C (hot washing—in the following: HW), and caustic soda at 80°C with water containing 2 m% NaOH and CTAB at a concentration of 9.2 mm (detergent washing—in the following: DW). A solid‐liquid ratio of 50 g plastic on 1 L washing medium was applied for each washing experiment. Plastics were stirred using an agitator at a speed of 200 rotations per minute. The washing medium was heated up to the desired temperature of 25°C or 80°C through a heating mantle. The washing time was 10 min for each washing procedure. Following washing, the plastics were rinsed with water at 25°C to eliminate any leftover detergents, and then placed in a desiccator to dry at room temperature for 24 h. Plastics were stored for a maximum of two weeks at 0°C before analysis.

### 2.3 Chemicals

Anhydrous sodium sulfate and dichloromethane (DCM) were obtained from Th. Geyer, Renningen, Germany, and liquid nitrogen were purchased from Linde GmbH, Pullach, Germany. DCM was freshly distilled prior to use. A series of alkanes from n‐hexane to n‐triacontane (Fluka, Steinheim, Germany, and Sigma–Aldrich, Steinheim, Germany) was used to determine linear retention indices (RIs).

The following reference compounds were purchased from the suppliers shown (commercial and commonly used names are given when applicable): ethyl 2‐methylbutanoate ≥ 98%; 1,8‐cineole ≥ 99%; 1‐octen‐3‐one ≥ 90%; rose oxide ≥ 90%; tetrahydrolinalool ≥ 97%; dihydromyrcenol ≥ 99%; linalool ≥ 97%; α‐damascone ≥ 99%; α‐isomethylionone ≥ 95%; β‐ionone ≥ 96%; eugenol ≥ 99%; 2‐methoxynaphthalene ≥ 99%; hexyl salicylate ≥ 99%; decanoic acid ≥ 98%; α‐hexylcinnamaldehyde ≥ 95%; 3‐phenylpropanoic acid ≥ 99%; γ‐terpinene ≥ 97%; acetic acid ≥ 99%; camphor ≥ 95%; acetophenone ≥ 98%; 2,4,6‐trichloroanisole ≥ 99%; benzothiazole ≥ 96%; γ‐nonalactone ≥ 98%; γ‐decalactone ≥ 98%; (trans)‐4,5‐epoxy‐(E)‐dec‐2‐enal ≥ 97% (aromaLAB AG, Freising, Germany); cis‐3‐hexenyl salicylate of unknown purity (Astatech, Pennsylvania, USA); propyl 2‐methylbutanoate of unknown purity (Symrise AG, Holzminden, Germany); vanillin ≥ 99% (abcr GmbH & Co. KG, Karlsruhe, Germany); 3a,4,5,6,7,7a‐hexahydro‐4,7‐methano‐1H‐inden‐6‐yl acetate (verdyl acetate) ≥ 97%; 1‐(1,2,3,4,5,6,7,8‐octahydro‐2,3,8,8,‐tetramethyl‐2‐naphthyl)ethan‐1‐one (methylcyclomyrcetone) ≥ 94% (Essencia AG, Winterthur, Switzerland).

### 2.4 Sensory Evaluation

#### 2.4.1 Trained Sensory Panel

Panelists were trained assessors from the Chair of Aroma and Smell Research (Department of Chemistry and Pharmacy—FAU, Erlangen, Germany). Five female and three male panelists (age range: 23–35 years) participated in the sensory evaluation of all plastic samples. Before participation in the experiments, panelists were trained during weekly sessions with selected odor solutions to identify and name odorants correctly.

#### 2.4.2 Procedure

The sensory evaluation was carried out in a room designed for this purpose, located at the Chair of Aroma and Smell Research (FAU, Erlangen, Germany). For the descriptive evaluation together with odor intensity ratings, 15 g (±0.1) of the sample were presented to the panel inside a 140 mL covered glass vessel. For each sample, a code was assigned.

The odor profile analyses were carried out in three phases according to instructions provided in ISO‐13299 [17]. In the first phase, the sensory impressions were individually described by the panelists. After collecting all odor qualities, the panel agreed on the main attributes as well as on the respective odorant references, with approval from more than half of the panelists. Aqueous solutions of fruity, citric, soapy, and flowery smells were prepared with ethyl 2‐methylbutanoate, nonanal, decanal, and linalool, respectively. Actual pieces of cardboard paper were used as a reference for the odor attribute cardboard‐like [18], as panelists could not agree on references for the moldy smell. In the last phase, the panelists were asked to evaluate the intensities of these attributes, in parallel with the overall intensities of the samples on a scale from 0 (no perception) to 10 (strong perception). Additionally, the hedonic perception of the samples was evaluated on a scale from 0 (dislike) to 10 (like). Results as medians were plotted in a spider‐web diagram.

#### 2.4.3 Data Analysis

To evaluate differences between plastic treated with the different washing processes in respect to intensity and hedonic ratings, statistical analysis was done using SPSS (IBM, New York, USA) and Microsoft Excel software (Microsoft, Redmond, USA), applying the Kruskal‐Wallis test and Dunn‐Bonferroni post‐hoc tests (pairwise comparisons with significance values adjusted by Bonferroni correction).

### 2.5 Isolation of Volatiles

To extract volatile organic compounds (VOCs), 10 g (± 0.1 g) of shredded plastic was stirred in 250 mL of DCM for 30 min. After filtering, the volatile portion was separated from non‐volatile compounds using the Solvent Assisted Flavor Evaporation (SAFE) method as described by Engel and colleagues [19]. The distillation was carried out under high vacuum with the flask in a 50°C water bath and the SAFE apparatus set to 55°C. The distillate, collected into a liquid nitrogen‐cooled flask, was thawed at room temperature and dried over anhydrous sodium sulfate. After filtration, DCM was evaporated by Vigreux distillation to reduce the volume to 3 mL, then further concentrated to 0.1 mL via micro‐distillation.

### 2.6 Gas Chromatography‐Olfactometry (GC‐O)

GC‐O analysis was carried out according to previous studies conducted in the same laboratory [4, 16]. 2D gas chromatography with mass spectrometric and olfactometric detection (2D‐GC‐MS/O) was conducted using two Agilent 7890B systems (Agilent Technologies, Santa Clara, USA) coupled via a CTS 1 cryogenic trapping device (Gerstel GmbH & Co. KG). The second chromatographic dimension was interfaced with an Agilent 5977 Quadrupole MS. A 2 µL aliquot of each distillate was introduced using a Multipurpose Sampler MPS 2XL (Gerstel GmbH & Co. KG) in cold on‐column mode at an oven start temperature of 40°C.

For separation, a DB‐FFAP column (30 m × 0.32 mm, 0.25 µm film; J&W Scientific, Agilent Technologies GmbH) was employed in the first dimension and a DB‐5 column (30 m × 0.25 mm, 0.25 µm film; J&W Scientific, Agilent Technologies GmbH) in the second. Both ovens followed the same program: an initial hold at 40°C for 2 min, followed by a ramp of 8°C/min up to the final temperature (240°C, 10 min for DB‐FFAP; 250°C, 5 min for DB‐5).

The effluent from the first column was divided between an olfactory detection port (ODP, 270°C) and a flame ionization detector (FID, 250°C). Helium was used as carrier gas at 2.5 mL/min. Defined retention windows (∼0.4 min) were trapped at −100°C in a CTS system via the MCS2 multi‐column switching device (Gerstel GmbH & Co. KG). After thermal desorption at 250°C, analytes were transferred to the second GC. At the outlet of the DB‐5 column, the flow was split between the ODP (270°C) and the MS.

Mass spectra were acquired in EI mode at 70 eV over an m/z range of 35–400.

### 2.7 Odor Extract Dilution Analysis

Odor extract dilution analysis (OEDA) was performed to determine odor dilution (OD) factors of compounds [20] using stepwise distillate dilution with DCM (1:2 v/v). An FFAP column was employed for this process. GC‐O analyses used undiluted distillate (OD factor 1) and serial dilutions with OD factors of 3, 9, 27, 81, 243, 729, 2187, and 6561. Each compound's OD factor was determined as the highest dilution where its odor remained detectable at the sniffing port. Analyses continued until no odors were detected. As OD1 was revealed to be too highly loaded with odor impressions, the GC‐O analyses were started with OD3. The odor‐active areas of OD3 were additionally evaluated by a second trained person to ensure the detection of all odorants.

### 2.8 2D GC‐MS/O

The 2D‐GC‐MS/O analyses were carried out according to previous studies conducted in the same laboratory [9].

### 2.9 Identification Criteria of Odorants

For preliminary identification, the retention index (RI) and odor characteristics (O) of odor‐active compounds detected during GC‐O analyses with DB‐FFAP and DB‐5 capillary columns were compared to those of reference compounds (RC), which were selected from an internal odorant database. Subsequently, 2D‐GC–MS/O analysis was conducted to determine each molecular structure by matching the mass spectrum (MS) of the odorant with that of the corresponding RC. When a match was achieved, the odorant was confirmed via concordance of RI, O, and MS data [9].

## 3 Results and Discussion

### 3.1 Sensory Evaluation

#### 3.1.1 Hedonic Ratings, Intensity Ratings, and Odor Profiles

The Kruskal‐Wallis test for the hedonic ratings showed a statistically significant difference between the groups (H = 11.17, *p* = 0.011). Thus, the hedonic ratings differed significantly across the treatment groups (UW, RT, HW, DW). Post‐hoc tests indicated a significant difference between the UW vs. DW groups for hedonic ratings (z = −3, 24, *p* = 0.001). None of the other pairwise comparisons were statistically significant (*p* > 0.05).

**FIGURE 1:**
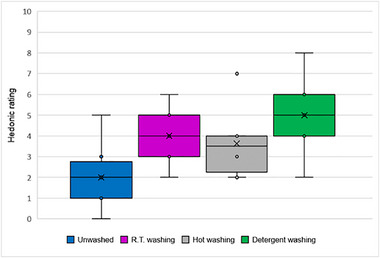
Overall hedonic ratings of PP before (UW) and after different washing processes (RT, HW, DW). Data were obtained from a panel of 8 participants. The scale is from 0 (dislike) to 10 (like). Boxplot data: lower whisker: minimum, higher whisker: maximum; dots: outliers; x: mean, dash: medians.

The lowest hedonic rating was obtained for the unwashed samples (Figure 1).

**FIGURE 2:**
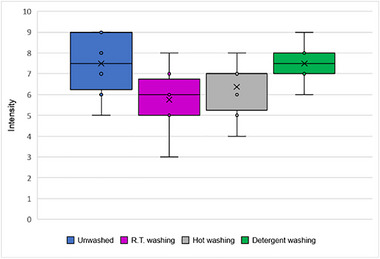
Overall odor intensities of PP before (UW) and after different washing processes (RT, HW, DW). Data were obtained from a panel of 8 participants. Scale is from 0 (no perception) to 10 (strong perception). Boxplot data: lower whisker: minimum, higher whisker: maximum; dots: outliers; x: mean, dash: medians.

The median hedonic ratings for PP samples vary notably across the different washing treatments, with the UW samples having the lowest median rating of 2. The DW method showed the most substantial improvement, with a median rating of 5. RT and HW resulted in median ratings of 4.0 and 3.5, respectively, both showing perceptual improvements over the unwashed samples. Intensity ratings for PP are depicted in Figure 2.

The Kruskal‐Wallis test revealed a significant difference between the groups (UW, RT, HW, DW) for odor intensity ratings (H = 8.19, *p* = 0.042). This result suggests that odor intensity ratings differ significantly between at least one of the groups. To further investigate these differences, Dunn‐Bonferroni post‐hoc pairwise comparisons were conducted. When conducted, none of the pairwise comparisons were statistically significant after applying the Bonferroni correction (*p* > 0.05).

In this context, it is important to mention that the Bonferroni correction is designed to control the family‐wise error rate by adjusting p‐values based on the number of comparisons [21]. However, this correction is very conservative, especially with smaller datasets, as in this current study (N = 32). This happens because the Bonferroni correction raises the threshold for statistical significance [21]. For example, instead of testing each pair at *α* = 0.05, each test is judged against a much smaller 𝛼 (in this study, 𝛼 ≈ 0.0125, because there are 4 comparisons), thus, only very big differences between groups will remain significant after the adjustment, which can explain why post‐hoc tests show no significant differences.

On PP intensity ratings, the UW and DW samples have the highest median ratings of 7.5, indicating that the DW washing method does not significantly reduce odor intensity compared to unwashed samples. RT washing resulted in a median odor intensity rating of 6, showing a modest reduction in odor intensity. HW washing showed a median of 7, which is slightly lower than the unwashed and detergent‐washed samples but still higher than the RT washed group. The Kruskal‐Wallis test did reveal an overall significant difference between the groups (*p* > 0.05).

**FIGURE 3:**
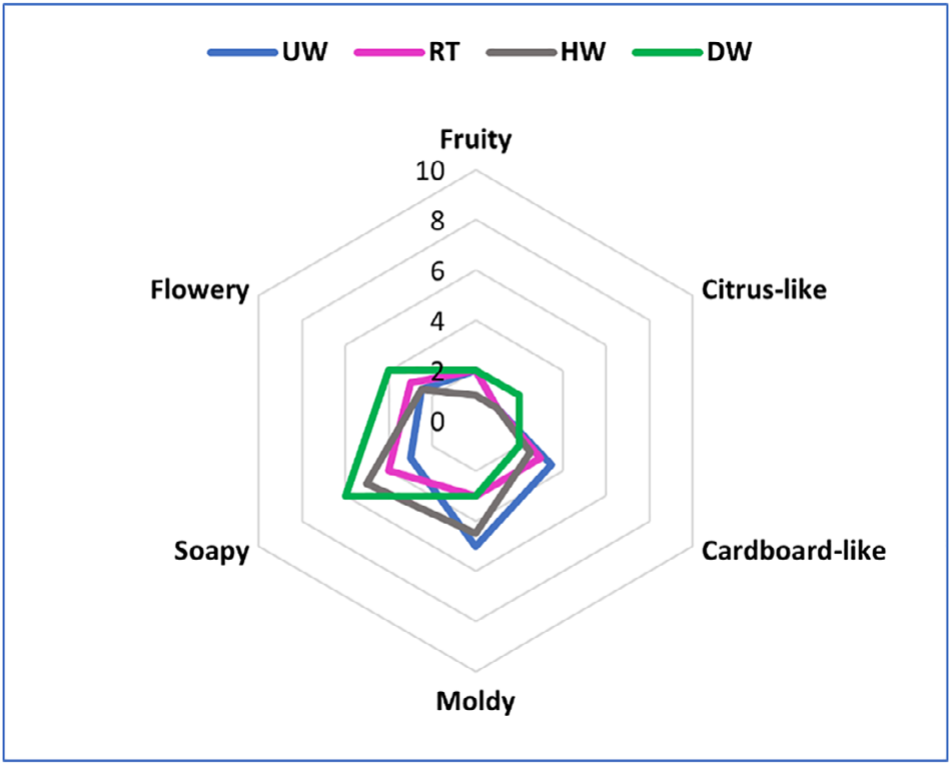
Odor profile of PP (right) before (UW) and after different washing processes (RT, HW, DW). Data are displayed as medians of intensity ratings from a panel of 8 participants. Scale is from 0 (no perception) to 10 (strong perception).

The odor profile analysis described the samples with the attributes fruity, citrus‐like, cardboard‐like, moldy, soapy, and flowery. The median intensity ratings determined for these attributes during the comparative odor profile analyses for PP are displayed in a spider web diagram (Figure 3).

The odor profile varied depending on the washing treatment. For the moldy attribute, the UW samples gave the highest intensity with a median of 5, while this intensity decreased to 3 in both RT and DW samples, and only slightly to 4.5 in HW samples. On the other hand, the soapy attribute had the highest intensity found in the DW samples, with a median of 6, compared to 3 in the UW samples. The HW samples also showed an increase in the soapy odor, with a median of 5, while the RT samples had a median of 4. For the flowery attribute, the DW samples again had the highest intensity, with a median of 4.0, compared to 2.5 in the UW samples. In contrast, the fruity and citrus‐like odors remained consistent across treatments, with the citrus‐like odor slightly increasing in the DW samples (2), while other treatments remained the same (1). Cardboard‐like attribute showed a decreasing trend with washing treatments, starting from a median of 3.5 in the UW samples and reducing to 2 in the DW samples.

The statistical analysis of the odor profile data from PP plastic subjected to various washing methods showed that most sensory attributes remained statistically similar (*p* > 0.05). When examining the fruity smell, no meaningful differences were detected among the treatment groups based on Kruskal‐Wallis testing, indicating that washing protocols did not significantly alter this particular olfactory property (H = 2.56, *p* = 0.465). The citrus‐like aroma followed a similar pattern, with no statistically meaningful variations observed between untreated (UW), room temperature (RT), hot water (HW), and detergent‐washed (DW) samples (H = 1.35, *p* = 0.717).

The perception of intensity for cardboard‐like odor also indicated no significant differences observed across the treatments (H = 1.53, *p* = 0.675). On the other hand, for the moldy attribute, the Kruskal‐Wallis test showed a significant difference (H = 7.20 and *𝑝* = 0.066 *p* = 0.066) when comparing all groups. However, no further significant differences were found between specific groups in post‐hoc comparisons (*p* > 0.05). Differently, washing treatments showed a significant difference for the soapy attribute (H = 14.08, *p* = 0.003). Specifically, post‐hoc Dunn‐Bonferroni tests revealed a significant difference between DW and UW samples (z = −2.73, *p* = 0.041), indicating that the detergent washing significantly increased the intensity of the soapy odor. Other pairwise comparisons were not significant: UW vs HW (z = −2.26, 𝑝 = 0.143 *p* = 0.143), RT vs HW (z = −1.42, 𝑝 = 0.942 *p* = 0.942), RT vs DW (z = −2.63, 𝑝 = 0.052 *p* = 0.052), and HW vs DW (z = −2.31, 𝑝 = 0.116 *p* = 0.116). Lastly, treatments showed no difference for the flowery attribute (H = 1.83, *p* = 0.608), further confirming that most odor attributes were not significantly affected by the washing treatments, except for a clear difference in the soapy odor between DW and UW samples.

### 3.2 Characterization of the Odorants

The GC‐O analyses led to an olfactometric detection of 32 odorants on a FFAP column (Table 1). The highest OD factors (6561) were assigned to verdyl acetate (fruity, banana‐like), methylcyclomyrcetone isomer (flowery), and eugenol (clove‐like). The compounds 1,8‐cineole (eucalyptus‐like), α‐isomethylionone (flowery), and β‐ionone (flowery) were detected with OD factors of 2187. All of these six substances were still present in washed samples; however, with lower or higher OD factors, depending on the substance.

**TABLE 1:** Odor‐active compounds identified in distillates obtained from different post‐consumer PP before and after the respective washing processes. Displayed are the OD factors obtained by cOEDA performed by GC‐O, as well as the respective identification criteria.

Compound	Odor quality	R.I.		OD‐factor				Identification criteria
		DB‐FFAP	DB‐5					
				UW	RT	HW	DW	
ethyl 2‐methylbutanoate	Fruity, strawberry‐like	1058	850	243	243	729	243	RI, OQ, MS, RC
propyl 2‐methylbutanoate	Fruity, pineapple‐like	1138	950	27	9	9	9	RI, OQ, MS, RC
unknown	Fuel‐like, rubber‐like	1160	—	243	243	243	243	
1,8‐cineole	Eucalyptus‐like	1198	1025	2187	729	2187	243	RI, OQ, MS, RC
unknown	Fruity, citrus‐like, ethereal	1234	—	81	81	243	81	
γ‐terpinene	Turpentine‐like, soapy	1245	1109	243	243	243	243	RI, OQ, MS, RC
1‐ octen‐3‐one	Mushroom	1291	985	81	81	243	81	RI, OQ, RC
rose oxide	Soapy, flowery	1333	1105	729	729	243	243	RI, OQ, MS, RC
tetrahydrolinalool	Flowery	1419	1091	243	243	243	243	RI, OQ, MS, RC
acetic acid	Vinegar‐like	1444	n.d.	27	27	27	27	RI, OQ, RC
dihydromyrcenol	Soapy, citrus‐like, flowery	1465	1068	729	729	81	27	RI, OQ, RC, MS
camphor	Eucalyptus‐like	1495	1150	81	81	243	81	RI, OQ, RC
linalool	Flowery	1536	1097	729	243	243	243	RI, OQ, MS, RC
acetophenone	Marzipan‐like, flowery	1634	1071	81	81	27	27	RI, OQ, RC
α‐damascone	Fruity, apple juice‐like	1772	1385	729	729	729	729	RI, OQ, MS, RC
2,4,6‐trichloroanisole	Cork‐like, musty	1801	1325	243	243	243	243	RI, OQ, RC
α‐isomethylionone	Flowery, rose‐like	1850	1493	2187	2187	2187	2187	RI, OQ, MS, RC
verdyl acetate	Fruity, banana‐like	1888	1448	6561	2187	2187	2187	RI, OQ, MS, RC
benzothiazole	Rubber‐like	1926	1230	81	81	81	81	RI, OQ, RC
β‐ionone	Flower, violet‐like	1929	1494	2187	2187	243	243	RI, OQ, MS, RC
(*trans*)‐4,5‐epoxy‐(*E*)‐dec‐2‐enal	Metallic	2008	1380	243	243	243	243	RI, OQ, RC
γ‐nonalactone	Coconut‐like	2024	1370	243	81	81	81	RI, OQ, MS, RC
methylcyclomyrcetone isomer	Flowery	2093	1698	6561	2187	243	2187	RI, OQ, MS, RC
γ‐decalactone	Fruity	2128	1439	243	243	243	243	RI, OQ, MS, RC
eugenol	Clove like	2156	1361	6561	2187	6561	2187	RI, OQ, MS, RC
2‐methoxynaphthalene	Glue‐like, almond‐like	2185	1463	243	243	6561	243	RI, OQ, MS, RC
hexyl salicylate	Soapy	2200	1686	243	243	243	243	RI, OQ, MS, RC
decanoic acid	Soapy	2255	1374	243	81	81	81	RI, OQ, MS, RC
*cis*‐3‐hexenyl salicylate	Soapy, flowery	2280	1665	729	243	6561	243	RI, OQ, MS, RC
α‐hexylcinnamaldehyde	Soapy, flowery	2360	1748	243	243	243	243	RI, OQ, MS, RC
vanillin	Vanilla like	2556	1401	729	243	729	243	RI, OQ, RC
3‐phenylpropanoic acid	Honey comb‐like	2553	1338	81	81	81	81	RI, OQ, RC

RI: retention index according to Van den Dool and Kratz (1963), OD: Odor dilution factor on capillary DB‐FFAP according to Grosch (2001), MS: mass spectrum, n.d.: not detected, O: odor quality perceived at the ODP, RC: comparison of the respective data with a reference compound. Only compounds with OD factors equal to or higher than 3 are depicted.

Out of the 32 odor‐active substances, 30 were identified (Table 1). In addition to the six already mentioned compounds, these were ethyl 2‐methylbutanoate (strawberry‐like, fruity), propyl 2‐methylbutanoate (fruity, pineapple‐like), 1,8‐ cineole (eucalyptus‐like), γ‐terpinene (turpentine‐like, soapy), 1‐octen‐3‐one (mushroom‐like), rose oxide (soapy, flowery), tetrahydrolinalool (flowery), acetic acid (vinegar‐like), dihydromyrcenol (soapy, citrus‐like, flowery), camphor (eucalyptus‐like), linalool (flowery), acetophenone (marzipan‐like, flowery), α‐damascone (fruity, apple juice‐like), 2,4,6‐trichloroanisole (musty), benzothiazole (rubber‐like), (*trans*)‐4,5‐epoxy‐(E)‐dec‐2‐enal (metallic), γ‐nonalactone (coconut‐like), γ‐decalactone (fruity), 2‐methoxynaphthalene (glue‐like, almond‐like), hexyl salicylate (soapy), decanoic acid (soapy), *cis*‐3‐hexenyl salicylate (soapy, flowery), α‐hexylcinnamaldehyde (soapy, flowery), vanillin (vanilla‐like) and 3‐phenylpropanoic acid (honey comb‐like).

**FIGURE 4:**
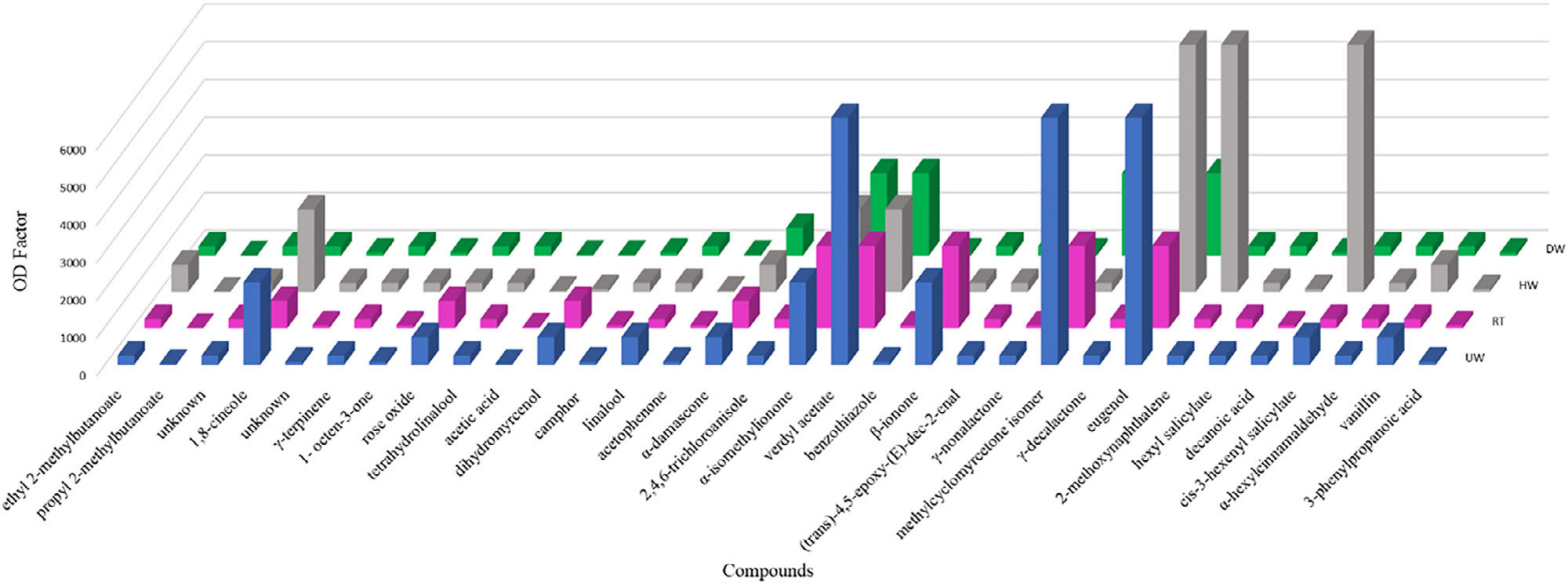
Overview comparison of intensity ratings from compounds identified in post‐consumer PP before and after washing processes.

The OD factors from unwashed samples showed some differences from those of the washed PP, as shown in Table 1 and Figure 4. For some substances, the OD factors in the washed PP material were lower than those observed for the unwashed PP. For example, for β‐ionone, the OD factor was diminished from 2187 (UW, RT) to 243 (HW, DW). However, the OD factor was maintained or even increased for other compounds, depending on which washing process was used. This was, for instance, the case for α‐isomethylionone (same OD factors across all treatments) and eugenol, which maintained the same OD factors when comparing UW and HW samples. Another case was for 2‐methoxynaphthalene, which greatly increased when comparing UW and HW samples (Table 1 and Figure 4).

### 3.3 Odorant Composition and New Components Found

A great number of odorants identified in this study have previously been described as constituents of plastic packaging waste and recycled material, and their potential origins have been discussed in detail elsewhere [10, 11, 16, 22].

To the best of our knowledge, the compounds rose oxide, dihydromyrcenol, hexyl salicylate, cis‐3‐hexenyl salicylate, and α‐hexylcinnamaldehyde are, however, reported here for the first time in post‐consumer PP rigid plastics. Dihydromyrcenol has previously been described when analyzing volatile compounds causing undesirable odors in PP recycled plastic resin [23]. The compound methylcyclomyrcetone was also described in the literature in LDPE bags from different collection systems, with high OD factors (>6561) [9]. On another observation, when evaluating odorant compounds of recycled automotive PP, Prado and colleagues tentatively identified 1,8‐cineole, acetophenone, benzothiazole, and eugenol [22], which we further confirmed in this current study.

Additionally, Strangl and colleagues [16] addressed the odor characterization of different recycled post‐consumer PP samples from the packaging sector, with corresponding odor attributes having been given as soapy and flowery, amongst others. The study also revealed compounds such as ethyl 2‐methylbutanoate, 1,8‐cineole, linalool, acetophenone, verdyl acetate, eugenol, and 2‐methoxynaphthalene. This goes in alignment with this report, as the majority of substances identified were compounds exhibiting a flowery smell or related to gourmand notes. Besides the previously mentioned compounds, substances in this report that relate to flowery smells and gourmand notes are, namely, rose oxide, tetrahydrolinalool, linalool, α‐isomethylionone, 1‐methyl‐β‐ionone, β‐ionone, methylcyclomyrcetone isomer, cis‐3‐hexenyl salicylate, α‐hexyl cinnamaldehyde, and 3‐phenylpropanoic acid.

### 3.4 Potential Origin of the Components

Due to its mechanical and chemical properties, PP materials are a suitable choice to serve as containers for household products such as detergents, cleaning products, cosmetics, shampoo, and soap. The presence of the listed compounds in the previous section and the fact that PP polymers are extensively used for shampoo, soap, cosmetics, and detergent containers correspond with the flowery and soapy smell attributes determined for the samples.

For example, rose oxide (soapy, flowery) is a monoterpene ether and is considered one of the most relevant fragrance substances widely used for the creation of rosy notes. The compounds α‐hexylcinnamaldehyde, as well as linalool and eugenol, are amongst 26 fragrance substances that were named almost 2000 times as ingredients in household detergents [24].

Similarly, the presence of 2‐methoxynaphtalene identified in PP is likely caused by the migration of such compounds from detergents and cleaning agents into the polymer. Likewise, β‐ionone is a typical perfuming ingredient, as is most of the rest of the odorants identified in the present study (e.g., α‐damascone, 1‐methyl‐β‐ionone, α‐isomethylionone). Thus, the migration of odorants into the packaging material can significantly contribute to the distinct odor of rigid PP waste and recycled material due to their high odor potencies.

### 3.5 Washing Efficiency and Change in Odor Profile

Strangl and colleagues reported having achieved a significant overall odor reduction when evaluating a hot washing step to decontaminate post‐consumer PP [16]. In the named study, the authors separated post‐consumer PP by its color. Considering total intensity ratings, our investigations show that in the case of rigid PP, there was no significant decrease in the overall odor observable when comparing UW PP and RT, DW, and HW PP. Thus, the practical impact of these changes obviously remains limited, with no single treatment demonstrating a clear and consistent advantage in reducing odor intensity. Overall, the DW method even appeared to intensify odor qualities such as soapy and flowery.

Nevertheless, a change in the odor profile was observed when comparing different washing processes. In UW PP, the highest intensity was observed for the moldy smell, whereas for HW and DT, it was soapy and flowery. In view of that observation, it is important to note that the compound 2,4,6‐trichloroanisole (cork‐like, musty) is a commonly potent contributor to moldy impressions [25, 26]. Thus, apart from this specific compound, it is suggested that the high intensity of the moldy attribute in UW PP can be due to the combined effect of several odorant compounds in the sample. Substances olfactometric detection was well in line with the results of sensory analysis, as α‐isomethylionone (flowery), methylcyclomyrcetone (flowery), and β‐ionone (flowery) were amongst the compounds with the highest OD factors, together with 1,8‐cineole (eucalyptus‐like), verdyl acetate (fruity, banana‐like), and eugenol (clove‐like).

In another instance, and using an electronic nose, Men and colleagues [27] demonstrated that elevating the ambient temperature with a heater leads to a rise in the odor intensity of volatile compounds released from 4 different types of automotive PP. At 50°C, the materials exhibited odor intensity values of approximately 1.3. When the temperature was increased to 80°C, these values rose to 1.8, 2.0, 2.3, and 1.9 across the four tested materials. The researchers' application of a mathematical model based on a component score coefficient matrix for the e‐nose does not clearly indicate whether those values are generally high or low from a human perspective. Nonetheless, these findings suggest a direct relationship between temperature elevation and an increase in odor intensity, an observation that we confirm in this report. For example, and interestingly, HW treatment increased the OD factor for 2‐methoxynaphthalene and cis‐3‐hexenyl salicylate, when compared to UW. This can be due to a higher permeability and migration effect of the compound from the plastic, induced by the high temperature, making it easier for absorbed compounds in the bulk of the plastic to migrate to the surface and thus be released, but such a hypothesis requires further research. A study for PE concluded that exposure to caustic solutions can promote thermo‐oxidative degradation, leading to chain scission, increased carbonyl formation, reduced molecular weight, and lower thermal and oxidative stability [7, 28]. These structural changes translate into higher melt‐flow rates and reduced performance during reprocessing. In contrast, PP appears far less sensitive to wash chemistry: cold and hot‐water washing removes surface contaminants and odors without measurable oxidation or polymer damage [29].

## 4 Conclusions

Focusing on post‐consumer rigid PP, the main objectives of the present study were to characterize odorous contaminants and to evaluate the overall odor reduction in the polymer after different washing processes. Overall, 32 compounds were detected by GC‐O. Identification with GC‐GC‐O/MS was achieved for 30 compounds in post‐consumer PP rigid plastics.

The sensory evaluations were in good agreement with the chemo‐analytical analysis, further confirming the contamination of post‐consumer PP with odorant compounds possibly coming from fragrances and food residues. There was, however, a change in the odor profile of the washed fractions. The application of different washing procedures led to a significant reduction in the moldy attribute, as well as a number of odorants and corresponding odor intensities, by applying sensory analysis, cOEDA, and GC‐O analyses. Additionally, depending on the type of washing process, some compounds appeared to increase in OD factors, ranging from 27 to 729, adding another challenge to the complexity of the task of polymer deodorization. Nevertheless, the washed PP polymers still showed a significantly strong smell as a consequence of odorous substances attached to the polymer, with intensities ranging from 7.5 to 5.5.

From an application perspective, the results suggest that washing alone does not reliably reduce overall odor intensity below perceptual thresholds required for high‐value packaging. While detergent washing altered odor character and improved hedonic perception, odor intensity remained >5.5 on a 0–10 scale. These findings indicate that (i) prior sorting of food and cosmetic packaging waste streams, (ii) limiting heavily perfumed input fractions, and (iii) implementing additional deodorization steps (e.g., degassing, reactive extrusion) are necessary for odor‐sensitive applications. The combination of sensory methods and cOEDA also provides a framework for quality‐control metrics to evaluate batch‐to‐batch odor variability during recycling.

In summary, our study confirms that the deodorization of post‐consumer PP rigid polymers still poses considerable challenges for the recycling industry. Additional approaches, such as prior sorting, are essential for comprehensive odor reduction to consequently ensure high‐quality recyclates and thus successfully move toward a circular economy for recycled PP polymers.

## Author Contributions

Tiago Belé: investigation, conceptualization, methodology, formal analysis, data‐curation, visualization, project administration, and writing – original draft. Martjn Roosen: conceptualization, methodology, writing – review, and editing. Helene M. Loos: conceptualization, methodology, project administration, supervision, writing – review and editing. Steven de Meester: funding acquisition, supervision, and writing – review and editing. Andrea Buettner: funding acquisition, project administration, supervision, and writing – review and editing.

## Funding

This project has received funding from the European Union's Horizon 2020 research and innovation programme under the Marie Sklodowska‐Cruie grant agreement No. 859885, and the German Research Foundation (Grant Number INST 90/979‐1 FUGG).

## Conflicts of Interest

The authors declare no conflicts of interest.

## Data Availability

The data that support the findings of this study are available from the corresponding author upon reasonable request.
